# Personality traits, gender roles and sexual behaviours of young adult males

**DOI:** 10.1186/s12991-016-0114-2

**Published:** 2016-10-21

**Authors:** Jacek Kurpisz, Monika Mak, Michał Lew-Starowicz, Krzysztof Nowosielski, Przemysław Bieńkowski, Robert Kowalczyk, Błażej Misiak, Dorota Frydecka, Jerzy Samochowiec

**Affiliations:** 1 Department of Psychiatry, Pomeranian Medical University, Szczecin, Poland; 2 Institute of Psychiatry and Neurology, 3rd Psychiatric Clinic, Warsaw, Poland; 3 Department of Sexology and Family Planning, Medical College in Sosnowiec, Sosnowiec, Poland; 4 Department of Obstetrics and Gynaecology, Specialistic Teaching Hospital, Tychy, Poland; 5 Institute of Psychiatry and Neurology, Warsaw, Poland; 6 Department of Sexology, Andrzej Frycz Modrzewski Cracow University, Kraków, Poland; 7 Department of Genetics, Wroclaw Medical University, Wrocław, Poland; 8 Department of Psychiatry, Wroclaw Medical University, Wrocław, Poland

**Keywords:** Sexual behaviour, Big Five personality traits, Gender roles

## Abstract

**Background:**

Previous studies have shown that personality characteristics affect sexual functioning. The aim of this exploratory study was to assess and describe the relationship between global personality traits and the stereotypical femininity and masculinity levels with the broad aspects of sexual behaviours and attitudes in the group of 97 heterosexual young adult men aged 19–39 and living in Poland.

**Methods:**

The ‘Big Five’ personality traits were measured with the NEO-FFI questionnaire; stereotypical femininity and masculinity with the Bem sex role inventory (BSRI); sexual disorders with the International index of erectile function (IIEF); socio-epidemiological data, sexual behaviours and attitudes towards sexuality with a self-constructed questionnaire.

**Results:**

We identified weak to moderate associations with particular sexual behaviours and attitudes. Neuroticism correlated positively with lower sexual satisfaction, self-acceptance and more negative attitudes towards sexuality; extraversion with higher desire, frequency of sexual intercourses, their diversity, sexual satisfaction, masculinity level and lower report of erectile problems; openness to experience with better quality of partnership, more positive attitudes towards sexual activity and masculinity level; conscientiousness with later sexual initiation age, more frequent and diverse sexual behaviours (but lower interest in masturbation and coitus interruptus), overall sexual satisfaction, satisfaction with one’s body and femininity level; agreeableness with a better quality of relationship with a partner, satisfaction from body, lower number of previous partners and more frequent sexual encounters (but less masturbation). Stereotypical masculinity, more so than femininity, was related to a wide range of positive aspects of sexuality.

**Conclusions:**

The Big Five personality traits and stereotypical femininity/masculinity dimensions were found to have a noticeable, but weak to moderate influence on sexual behaviour in young adult males.

## Background

There is a paucity of studies on relationships between personality structure and sexual functions, as well as expression of sexuality in the period of early adulthood [1, 2]. The quest for exploring the relationship between traits that describe human personality and sexual expression was initiated by Eysenck [3]. He used his 3-factor model (EPQ) to examine extraversion, neuroticism and psychoticism and describe patterns of sexual activity. He proposed to use ‘libido’ and ‘satisfaction’ scales to measure a number of continuums comprising various aspects of human sexual experience [4]. Other researchers suggested different theories and psychometric tools for measuring sexual-related traits as a separate entity, e.g. erotophobia-erotophilia scale [5] and self-monitoring scale [6]. Some researchers followed the idea of sexual-related traits in their studies, while others were still using the recognised personality taxonomies to investigate the field of sexuality [7]. In the latter group, the initial studies were based on Eysenck’s concept [3, 8], while the later ones shifted to exploit ‘The Big Five Model’ [3]. Discussion concerning a potential overlap and other relations to the Eysenck’s model can be found elsewhere [3, 9, 10]. In this study, we followed the ‘Big Five’ way of exploration.

According to Buss [11], the Big Five may capture some important features that represent individual differences in evolutionary strategies, which could be significantly related to engaging in specific sexual behaviours.

Previous studies, although limited in number, proved that existing personality taxonomies are indeed useful for explanation of some sexual attitudes and behaviours [3]. Shafer [4] states that global personality traits, such as the ‘Big Five’, have been shown to be moderate predictors of individual differences within sexuality.

We decided to verify that assumption, but in our study the main objective was to check if the ‘Big Five’ personality traits are related to engagement in particular sexual behaviours, occurrence of sexual problems and selected attitudes towards sexual expression.

Besides global personality traits, we hypothesised that in young adult men their perception of gender role may correlate with engagement in particular sexual activities and sexual expression. In brief, gender role is a social construct containing rules and characteristics (physical, emotional, intellectual) of stereotypically perceived femininity and masculinity [12, 13]. Under the pressure of social expectations, all members of society have to develop their personal gender role, as a part of their personality in the process of enculturation. S. L. Bem distinguished four gender role types, depending on the relationship of stereotypically masculine and feminine traits: masculine (high level of masculine and low of feminine traits), feminine (low level of masculine and high of feminine traits), androgynous (high levels of both masculine and feminine traits), or undifferentiated (low levels of both masculine and feminine traits; such individuals describe themselves differently than by using gender-related characteristics). Identification with a particular type affects the way that individuals construct their cognitions about the world and others.

In our study we decided to provide the answer to the following questions:

1. What is the relationship between the ‘Big Five’ characteristics, femininity and masculinity levels and tendency to engage in sexual behaviours like: penile-vaginal encounter, sexual activities without penetration, oral sex, anal sex, masturbation and mutual masturbation with partner, orgasm frequency and contraception usage?
2. What is the relationship between the ‘Big Five’ characteristics, femininity and masculinity levels and erectile functions?
3. What is the relationship between the ‘Big Five’ characteristics, femininity and masculinity levels and positive/negative attitudes towards sexual activity, satisfaction with sexual life, one’s body and self?
4. What is the relationship between the ‘Big Five’ characteristics, femininity and masculinity levels and relationship with a partner?
5. What is the relationship between the ‘Big Five’ characteristics, femininity and masculinity levels and sexual disorders report?

## Methods

### Participants

The 97 respondents were aged between 19 and 39 years (M = 29.28, SD = 5.83). The sample was composed of male students and workers of Pomeranian Medical University, patients of the occupational medicine centre, workers from a local building company, state office workers, public hospital staff and other volunteers who agreed to take part in the study. Participants were recruited between January 2014 and May 2015 in the city of Szczecin. Out of the total sample, 89 % were city dwellers and 11 % were rural residents. The majority of the sample had secondary (50 %) and college (39 %) education, which is quite a typical phenomenon in sexological studies [14]. About 75 % of the studied subjects were in a relationship. In regard to religion, 66 % identified themselves as Catholics, 29 % as non-believers and 3 % as ‘other’. Socio-demographic characteristics of the sample are presented in Table 1.

**Table 1:** Socio-demographic characteristics of the sample (*n* = 97)

	*n*	%
*Education*		
Elementary	10	10
Secondary	48	50
College	38	39
No answer	1	1
*Residency*		
City	86	86
Country	10	10
No answer	4	4
*Relationship*		
Single	20	21
In relationship	74	76
No answer	3	3
*Religion*		
Catholic	64	66
Other	3	3
Non-believer	28	29
No answer	2	2

### Procedure

The participants were selected according to age, to gather a relatively representative group for Polish young adults (basing on national census from 2012 [15]). Because of the expected problems which commonly follow sexological studies during the development of the study design, we decided not to use any additional criterion for the recruitment process. We have made such decision with awareness of its limitation to avoid the influence of taboo concerning information about sexual life in the Polish society, which could result in a great decrease in response rate.

In this paper, we decided to apply the quota sampling method. The respondents were qualified to sub-groups by age: 19–24 years (27 % of the sample), 25–29 years (25 % of the sample), 30–34 years (25 % of the sample) and 35–39 years (23 % of the sample). After giving their consent to take part in the study, the participants were given a paper-and-pencil version of the questionnaires to fill in at home and send back anonymously in the provided envelope with a stamp and address. They were provided with all the necessary instructions about the study, as well as the study procedure. Recruitment to the study was continued until a representative group of 100 respondents was gathered. The response rate was 36.7 %.

Men who reported being treated for sexual dysfunction at the time of the study or suffered from a serious somatic illness, mental illness or disabilities were excluded from the study. We decided to apply such criteria to exclude the possibility of sexual expression being in any way affected by such external causes. Our intention was to assess the exclusive impact of personality and gender characteristics on sexual behaviour and attitudes.

During the data analysis phase, we decided to exclude three non-heterosexual persons from the sample to make it more homogenous in relation to sexual orientation.

### Measures

Personality traits were assessed by the Polish version of NEO-FFI, a 60-item Big Five inventory. The Cronbach’s alpha reliability coefficients were as follows: 0.80 for neuroticism, 0.77 for extraversion, 0.82 for conscientiousness, 0.68 for agreeableness and 0.68 for openness to experience [16].

Masculinity-Femininity and gender roles were evaluated with the Polish version of the Bem sex role inventory (BSRI) [12, 17]. In this 35-item questionnaire the respondents were asked to assess on a scale from 1 (I strongly disagree) to 5 (I strongly agree) the extent to which given adjectives relating to stereotypical femininity and masculinity described them personally. The Cronbach’s alpha reliability coefficients were 0.78 for masculinity scale and 0.79 for femininity scale.

Sexual function was evaluated using a 15-question standardised and validated Polish version of the international index of erectile function (IIEF) questionnaire, which measures five domains of sexual functions in men: erectile and orgasmic functions, sexual desire, intercourse satisfaction, and overall satisfaction. Individuals who scored 26 or more points in the erectile function scale (EF) were considered as having normal erectile function. Mild dysfunction was diagnosed in patients with 22–25 point score, mild to moderate, 17–21, moderate, 11–16, and severe, 10 or less. Orgasmic function (OF), sexual desire (SD) and overall satisfaction (OS) were considered normal in patients with a score of 9 or more, whereas intercourse satisfaction (IS) was considered decreased in those with a score of 12 or less [18, 19]. Cronbach’s alpha range for the IIEF scales was from 0.73 to 0.99.

For socio-epidemiological data assessment we used a self-constructed questionnaire. It included questions concerning frequency of sexual behaviours such as condom usage, coitus interruptus on scales from 1-never to 5-always. Other questions measured the respondent’s and their partner’s perceived attitudes towards sexuality, quality of the relationship with a current sexual partner, satisfaction with sex life, satisfaction with one’s body, with self as a man, and self-esteem (on Likert’s scales from 1-very low level/bad to 5-very high level/very good). Religiosity type was assessed through a question asking for self-identification (catholic/other/non-believer) and religiosity level was measured on a scale based on a single question, ranging from 1-totally not religious to 5-very religious.

Sexual activity was defined as any of the following: caressing, foreplay, masturbation, vaginal or anal intercourse, or oral sex (declared mean number per month). There were also yes/no questions about pornography usage and staying in a relationship.

Frequency of sexual problems, such as erectile dysfunction, premature ejaculation, and delayed ejaculation were measured on scales from 1 (never) to 4 (almost always). All questions concerning sexual problems considered the period of the last 3 months.

### Statistical methods

For a statistical evaluation, we have chosen correlation and quasi-experimental plans. We used Pearson’s r and Spearman’s rho for the particular correlation analysis. For group comparisons we utilised the Kruskal–Wallis and Mann–Whitney tests because of their inequality. Additionally, we used the Bonferroni correction to highlight the strongest relationships, although we decided to set p value on a 0.05 level to avoid type II error. We checked the normality of variable distribution with the Shapiro–Wilk test.

Before we started the statistical analysis, the variables assessing frequency of particular sexual behaviours (sexual activity per month) were modified from continuous to discrete form of 10 equal groups, according to the percentile distribution observed in our sample. Such procedure was applied to avoid any possible bias related to the continuous form of the primary variables.

## Results

Table 2 shows statistical characteristics of the sexual behaviours in the sample. It proves that the studied sample presented a great individual diversity of these measures.

**Table 2:** Characteristics of the sexual behaviour in the sample (n = 97)

	Mean score	SE	SD
Sexual initiation (age)	17.79	0.26	2.47
Number of previous sexual partners	5.97	0.53	5.16
No. of sexual encounters per month (penile-vaginal)	11.90	0.88	8.52
No. of sexual activities without penetration per month	6.33	0.90	8.76
No. of anal sex per month	2.73	0.64	6.14
No. of oral sex per month	5.25	0.69	6.57
No. of mutual masturbation with partner per month	4.91	0.73	7.15
No. of masturbation per month	5.26	0.74	7.11
No. of orgasms per month	15.44	1.01	9.81
No. of any sexual activity per month	14.73	0.96	9.24
No. of satisfying sexual activities per month	12.60	0.98	9.22
Mean time of ejaculation latency	19.40	1.43	13.66

Variables are shown in a primary form, as continuous ones

Table 3 depicts statistical characteristics of the ‘Big Five’ traits in the investigated group. None of the measured traits in the sample has met close to normal distribution in the Shapiro–Wilk test.

**Table 3:** The ‘Big Five’ statistical description (n = 97)

	Min	max	Mean score	SE	SD
N	1	8	4.41	0.21	2.11
E	1	10	5.88	0.21	2.05
O	1	10	4.86	0.21	2.03
C	1	10	6.74	0.22	2.17
A	1	10	5.81	0.20	1.99

Table 4 shows description of the gender role measures. In the Shapiro–Wilk test, masculinity scores did not reach close to normal distribution, while femininity did [W(95) = 0.98, *p* > 0.05]. The dominating gender role type was stereotypically masculine, then androgynous, undifferentiated, with the feminine being the rarest.

**Table 4:** Femininity, masculinity and gender roles prevalence in the sample (n = 95)

	Mean score	SE	SD
F	51.85	0.79	7.70
M	52.71	0.85	8.32

Gender role type prevalence			
Masculine (%)	Feminine (%)	Androgynous (%)	Undifferentiated (%)
43	4	32	20

*F* stereotypical femininity, *M* stereotypical masculinity. Gender role types: Masculine low F, high M; Feminine high F, low M; Androgynous high both F and M; Undifferentiated low both F and M

Description of the sexual functioning (IIEF scores) is shown in Table 5, while prevalence of sexual problems is presented in Table 6. As expected, the studied sample was relatively sexually healthy.

**Table 5:** IIEF scores—statistical description

	Mean score	SE	SD
EF (*n* = 88)	27.20	0.37	3.43
OF (*n* = 88)	9.11	0.16	1.45
SD (*n* = 96)	7.74	0.16	1.56
IS (*n* = 88)	12.39	0.21	1.93
OS (*n* = 88)	9.10	0.14	1.28

Eight subjects were not sexually active in the past 4 weeks so only SD was measured for them. 1 response was lacking

*EF* erectile function, *OF* orgasmic function, *SD* sexual desire, *IS* intercourse satisfaction, *OS* overall satisfaction

**Table 6:** Sexual problems frequency report (*n* = 97)

	Never (%)	Sometimes (%)	Less than in a half of sexual encounters (%)	Almost always (%)
Erectile dysfunction	74	26	0	0
Premature ejaculation	59	34	4	3
Delayed ejaculation	74	25	0	1

In relation to age in the studied sample of males, we indicated significant correlations with masculinity level (*r* = −0.26; *p* < 0.05), number of previous partners (*r* = 0.24; *p* < 0.05), education level (*r*_s_ = 0.35; *p* < 0.001), condom usage (*r*_s_ = −0.25; *p* < 0.05), frequency of sexual encounters without penetration (*r*_s_ = −0.40; *p* < 0.001), anal sex (*r*_s_ = 0.29; *p* < 0.01), oral sex (*r*_s_ = −0.28; *p* < 0.01), and masturbation (*r*_s_ = −0.35; *p* < 0.01). The studied age sub-group comparison has revealed statistically significant differences in frequency of sexual activities without penetration, anal sex, oral sex and masturbation per month. There were also differences in satisfaction with one’s body. These findings are presented in Table 7.

**Table 7:** The age sub-groups (19–24, 25–29, 30–34, 35–39) comparison for measured variables

	Df	Chi^2^	*p* value	
Masculinity	3	12.07	<0.01	19–24 > 25–29 > 30–34 > 35-39
Education	3	16.72	<0.001^a^	19–24 < 25–29, 35–39 < 30–34
No. of sexual activities without penetration per month	3	15.16	<0.01	19–24 > 25–29 > 30–34 > 35–39
No. of anal sex per month	3	8.82	<0.05	19–24 < 25–29 < 30–34 < 35–39
No. of oral sex per month	3	11.51	<0.01	19–24 > 30–34 > 25–29 > 35–39
No. of masturbation per month	3	12.45	<0.01	19–24 > 25–29 > 30–34 > 35–39
Satisfaction with one’s body	3	8.01	<0.05	19–24 < 25–29 < 30–34 < 35–39

There are presented only these variables which had *p* < 0.05 in the Kruskal–Wallis test (all the variables measured in the study were tested). The last column shows relations of the variable levels between groups

^a^Means passing requirements of the Bonferroni correction (*p* < 0.0013158)

With reference to the ‘Big Five’ traits, Neuroticism was significantly negatively correlated with conscientiousness (*r* = −0.59; *p* < 0.001) and agreeableness (*r* = −0.25; *p* < 0.01). In the IIEF scales, it was correlated only with OS (*r* = −0.23; *p* < 0.05). There was no significant correlation with BSRI femininity or masculinity, nor with any sexual disorders. Among sexual behaviours, Neuroticism was negatively associated with anal sex (*r*_s_ = −0.39; *p* < 0.001) and positively with condom usage (*r*_s_ = 0.23; *p* < 0.05), as well as coitus interruptus (*r*_s_ = 0.26; *p* < 0.05). Regarding the attitudes, neuroticism was negatively correlated with satisfaction with one’s body (*r*_s_ = −0.52; *p* < 0.001), satisfaction with self as a man (*r*_s_ = −0.26; *p* < 0.01), satisfaction with sex life (*r*_s_ = −0.22; *p* < 0.05), one’s attitude toward sexual activity (*r*_s_ = −0.24; *p* < 0.05), partner’s attitude towards sexual activity (*r*_s_ = −0.22; *p* < 0.05), and quality of relationship with a partner (*r*_s_ = −0.23; *p* < 0.05).

Extraversion was found to be correlated with openness to experience (*r* = 0.30; *p* < 0.01). Among IIEF facets, extraversion was associated only with SD (*r* = 0.21; *p* < 0.05). In the field of sexual problems, there was a negative correlation with erectile dysfunction (*r*_s_ = −0.23; *p* < 0.05). Notably, there was a medium correlation with the BSRI dimension of masculinity (*r* = 0.39; *p* < 0.001). In the area of sexual behaviours, extraversion was correlated with sex per month (*r*_s_ = 0.34; *p* < 0.01), oral sex per month (*r*_s_ = 0.31; *p* < 0.01), orgasms per month (r_s_ = 0.26;* p* < 0.05), any sexual activity per month (*r*_s_ = 0.24; *p* < 0.05), and satisfying intercourses per month (*r*_s_ = 0.43; *p* < 0.001). Extraversion was also correlated with satisfaction with sex life (*r*_s_ = 0.22; *p* < 0.05). There was no significant correlation with personal attitudes, demographic features or other sexual characteristics.

Openness to experience was positively associated with extraversion and negatively with conscientiousness (*r* = −0.22; *p* < 0.05). There was no correlation with IIEF facets. Among the BSRI dimensions, Openness to experience was connected with masculinity (*r* = 0.21; *p* < 0.05). Surprisingly, neither the investigated sexual behaviours per month or demographic characteristics, nor personal attitudes were associated with this personality feature. However, Openness to experience correlated with the positive quality of relationship with a partner (*r*_s_ = 0.28; *p* < 0.01), partner’s positive attitude toward sexual activity (*r*_s_ = 0.34; *p* < 0.01) and satisfaction with sexual life (*r*_s_ = 0.32; *p* < 0.01).

Conscientiousness, apart from inverse correlations with Neuroticism (*r* = −0.59; p < 0.001) and openness to experience (*r* = −0.22; *p* < 0.05), was also positively associated with agreeableness (*r* = 0.45; *p* < 0.001). It correlated with OS (*r* = 0.28; *p* < 0.01) and BSRI femininity (*r* = 0.26; *p* < 0.01). Conscientiousness was on the one hand associated with later sexual initiation age (*r* = 0.24; *p* < 0.05), but on the other with more frequent sexual intercourse per month (*r*_s_ = 0.27; *p* < 0.01), anal sex per month (*r*_s_ = 0.28; *p* < 0.01), satisfying intercourses per month (*r*_s_ = 0.31; *p* < 0.01) and negatively with masturbation per month (*r*_s_ = −0.30; *p* < 0.01). None of the investigated demographic characteristics were associated with this personality feature. Conscientiousness was correlated with less frequent engaging in coitus interruptus (*r*_s_ = −0.36; *p* < 0.001) and premature ejaculation (*r*_s=_−0.22; *p* < 0.05). Regarding personal attitudes, there were correlations with satisfaction with one’s body (*r*_s_ = 0.43; *p* < 0.001).

Agreeableness, as previously mentioned, was inversely associated with neuroticism and conscientiousness. It was not connected with any of the IIEF facets. In reference to BSRI dimensions, Agreeableness positively correlated with femininity (*r* = 0.25; *p* < 0.05) but negatively with masculinity (*r* = −0.22; *p* < 0.05). It was also negatively associated with the number of previous sexual partners (r = −0.28; *p* < 0.01). As regards sexual behaviours, agreeableness was connected with engaging in sexual intercourse per month (*r*_s_ = 0.25; *p* < 0.05), mutual masturbation with partner per month (*r*_s_ = 0.21; *p* < 0.05) and negatively with masturbation (*r*_s_ = −0.25; *p* < 0.05). None of the investigated demographic characteristics were associated with this personality feature. Agreeableness correlated with satisfaction with one’s body (*r*_s_ = 0.25; *p* < 0.01), self-acceptance (*r*_s_ = 0.20; *p* < 0.05), good relationship with a sexual partner (*r*_s_ = 0.26; *p* < 0.05) and negatively with frequency of premature ejaculation (*r*_s_ = −0.24; *p* < 0.05).

All the findings concerning associations between the ‘Big Five’ traits and the other investigated variables are presented in Table 8.

**Table 8:** Correlations between the Big Five traits and other variables

Big Five domain	Neuroticism		Extraversion		Openness to experience		Conscientiousness		Agreeableness	
	*r*	*p*	*r*	*p*	*r*	*p*	*r*	*p*	*r*	*p*
EF (*n* = 88)	−0.17	NS	0.18	NS	−0.12	NS	0.05	NS	−0.13	NS
OF (*n* = 88)	−0.08	NS	0.01	NS	0.11	NS	−0.07	NS	−0.13	NS
SD (*n* = 96)	0.07	NS	0.21*	<0.05	0.16	NS	−0.10	NS	−0.14	NS
IS (*n* = 88)	−0.02	NS	0.09	NS	−0.03	NS	0.05	NS	−0.14	NS
OS (*n* = 88)	−0.23*	<0.05	0.03	NS	0.03	NS	0.28**	<0.01	0.04	NS
Femininity (*n* = 97)	−0.13	NS	0.09	NS	0.01	NS	0.26**	<0.01	0.25*	<0.05
Masculinity (*n* = 97)	−0.19	NS	0.39***^a^	<0.001	0.21*	<0.05	0.18	NS	−0.22*	<0.05
Sexual initiation (age) (*n* = 92)	−0.15	NS	−0.05	NS	−0.12	NS	0.24*	<0.05	0.19	NS
Time needed to ejaculate (min) (*n* = 91)	−0.11	NS	0.01	NS	0.02	NS	0.08	NS	−0.12	NS
Number of previous sexual partners (*n* = 96)	0.11	NS	0.18	NS	0.10	NS	−0.17	NS	−0.28**	<0.01
No. of sexual encounters per month (*n* = 82)	−0.11	NS	0.34**	<0.01	0.12	NS	0.27*	<0.05	0.25*	<0.05
No. of sexual activities without penetration per month (*n* = 95)	0.26	<0.05	0.19	NS	0.18	NS	−0.22*	<0.05	−0.15	NS
No. of anal sex per month (*n* = 93)	−0.39***^a^	<0.001	−0.07	NS	−0.05	NS	0.28**	<0.01	0.19	NS
No. of oral sex per month (*n* = 92)	0.10	NS	0.31**	<0.01	0.17	NS	−0.01	NS	−0.05	NS
No. of mutual masturbation with partner per month (*n* = 95)	−0.10	NS	0.10	NS	−0.04	NS	0.12	NS	0.21*	<0.05
No. of masturbation per month (*n* = 93)	0.04	NS	−0.05	NS	0.07	NS	−0.30**	<0.01	−0.25*	<0.05
No. of orgasms per month (*n* = 94)	−0.07	NS	0.26*	<0.05	0.17	NS	0.10	NS	0.02	NS
No. of any sexual activity per month (*n* = 93)	−0.17	NS	0.24*	<0.05	0.10	NS	0.24*	<0.05	0.14	NS
No. of satisfying sexual activities per month (*n* = 89)	−0.20	NS	0.43***^a^	<0.001	0.09	NS	0.31**	<0.01	0.19	NS
Condom usage frequency (*n* = 94)	0.23*	<0.05	−0.02	NS	−0.08	NS	−0.03	NS	−0.09	NS
Coitus interruptus frequency (*n* = 94)	0.26*	<0.05	0.18	NS	0.08	NS	−0.36***	<0.001	−0.20	NS
Satisfaction with one’s body (*n* = 96)	−0.52***^a^	<0.001	0.08	NS	−0.01	NS	0.43***^a^	<0.001	0.26**	<0.01
Satisfaction with self as a man (*n* = 97)	−0.26**	<0.01	0.18	NS	0.05	NS	0.13	NS	0.05	NS
Satisfaction with sexual life (*n* = 95)	−0.22*	<0.05	0.23*	<0.05	0.32**	<0.01	0.15	NS	0.12	NS
Self-acceptance (*n* = 97)	−0.08	NS	0.01	NS	0.12	NS	0.17	NS	0.20*	<0.05
Quality of relationship with a partner (*n* = 92)	−0.23*	<0.05	0.15	NS	0.28**	<0.01	0.19	NS	0.26*	<0.05
One’s attitude toward sexual activity (*n* = 97)	−0.22*	<0.05	0.14	NS	0.20	<0.05	0.17	NS	0.06	NS
Partner’s attitude toward sexual activity sex (*n* = 91)	−0.25*	<0.05	0.10	NS	0.34**	<0.01	0.19	NS	0.09	NS
Erectile dysfunction (*n* = 95)	0.03	NS	−0.23*	<0.05	−0.02	NS	−0.19	NS	−0.02	NS
Premature ejaculation (*n* = 95)	0.06	NS	−0.07	NS	−0.04	NS	−0.22*	<0.05	−0.25*	<0.05
Delayed ejaculation (*n* = 95)	−0.06	NS	0.08	NS	0.01	NS	−0.15	NS	−0.08	NS

The table shows correlations with the IIEF scales, stereotypical femininity and masculinity from the BSRI, frequency of sexual behaviours, measured attitudes and declared sexual problems. Different numbers of n for particular comparisons mean lack of given answer. Pearson’s *r* was used for: the IIEF and BSRI scales, sexual initiation age and time needed to ejaculate. Spearman’s rho was used for: frequency of sexual behaviours, attitudes and declared sexual problems

IIEF abbreviations: *EF* erectile function, *OF* orgasmic function, *SD* sexual desire, *IS* intercourse satisfaction, *OS* overall satisfaction

* *p* < 0.06, ** *p* < 0.01, *** *p* < 0.001

^a^Means passing requirements of the Bonferroni correction (*p* < 0.0003226). In the IIEF eight subjects submitted that they were not sexually active in the past 4 weeks, so only SD was measured for them

In our study, BSRI stereotypical femininity dimension correlated with EF (*r* = 0.27; *p* < 0.05) and OF (*r* = 0.22; *p* < 0.05), time needed to ejaculate in minutes (*r* = 0.33; *p* < 0.01), anal sex per month (*r* = 0.28; *p* < 0.01) and, surprisingly, with masculinity (*r* = 0.31; *p* < 0.01).

Stereotypical masculinity additionally correlated with EF (*r* = 0.30; *p* < 0.01), SD (*r* = 0.29; *p* < 0.01), age (*r* = −0.26; *p* < 0.01), number of previous sexual partners (*r* = 0.21; *p* < 0.05), sex without penetration per month (*r*_s_ = 0.35; *p* < 0.001), orgasms per month (*r*_s_ = 0.32; *p* < 0.01), any sexual activity per month (*r*_s_ = 0.31; *p* < 0.01), satisfying sexual intercourses per month (*r*_s_ = 0.25; *p* < 0.05), satisfaction with sex life (*r*_s_ = 0.30; *p* < 0.01) lower erectile dysfunction frequency (*r*_s_ = −0.31; *p* < 0.01), one’s better attitude toward sexual activity (*r*_s_ = 0.30; *p* < 0.05), partner’s attitude toward sexual activity (*r*_s_ = 0.21; *p* < 0.05), satisfaction with sexual life (*r*_s_ = 0.30; *p* < 0.05), partner’s attitude toward sexual activity (*r*_s_ = 0.25; *p* < 0.05) and femininity.

All the investigated associations between BSRI dimensions and other variables are depicted in Table 9.

**Table 9:** Correlations between the BSRI femininity, masculinity and other variables

BSRI	Femininity		Masculinity	
	*r*	*p*	*r*	*p*
EF (*n* = 86)	0.30*	<0.05	0.29**	<0.01
OF (*n* = 86)	0.22*	<0.05	0.07	NS
SD (*n* = 94)	0.04	NS	0.28**	<0.01
IS (*n* = 85)	0.18	NS	0.18	NS
OS (*n* = 86)	0.12	NS	0.19	NS
Sexual initiation (age) (*n* = 90)	−0.08	NS	−0.21	NS
Time needed to ejaculate (min) (*n* = 89)	0.33**	<0.01	0.15	NS
Number of previous sexual partners (*n* = 94)	0.06	NS	0.21*	<0.05
No of Sexual encounters per month (*n* = 81)	−0.04	NS	0.16	NS
No of sexual activities without penetration per month (*n* = 81)	−0.10	NS	0.35***	<0.001
No of anal sex per month (*n* = 91)	0.28**	<0.01	−0.13	NS
No of oral sex per month (*n* = 90)	−0.19	NS	0.13	NS
No of mutual masturbation with partner per month (*n* = 93)	0.17	NS	−0.12	NS
No of masturbation per month (*n* = 91)	−0.12	NS	0.15	NS
No of orgasms per month (*n* = 92)	0.02	NS	0.32**	<0.01
No of any sexual activity per month (*n* = 91)	0.09	NS	0.31**	<0.01
No of satisfying sexual activities per month (*n* = 87)	0.02	NS	0.25*	<0.05
Condom usage frequency (*n* = 92)	−0.07	NS	0.12	NS
Coitus interruptus frequency (*n* = 92)	−0.06	NS	−0.10	NS
Satisfaction with one’s body (*n* = 94)	0.13	NS	0.16	NS
Satisfaction with self as a man (*n* = 95)	0.10	NS	0.13	NS
Satisfaction with sex life (*n* = 93)	0.03	NS	0.30**	<0.01
Self-acceptance (*n* = 95)	0.05	NS	−0.18	NS
Quality of relationship with a partner (*n* = 90)	−0.04	NS	0.20	NS
One’s attitude toward sexual activity (*n* = 95)	0.04	NS	0.30**	<0.01
Partner’s attitude toward sexual activity (*n* = 89)	−0.07	NS	0.25*	<0.05
Erectile dysfunction (*n* = 95)	−0.16	NS	−0.31**	<0.01
Premature ejaculation (*n* = 95)	0.05	NS	0.05	NS
Delayed ejaculation (*n* = 95)	−0.01	NS	0.14	NS

The table shows correlations with IIEF scales, frequency of sexual behaviours, measured attitudes and declared sexual problems. Different numbers of n for particular comparisons mean lack of given answer. Pearson’s *r* was used for: IIEF scales, sexual initiation age and time needed to ejaculate. Spearman’s rho was used for: frequency of sexual behaviours, attitudes and declared sexual problems. * *p* < 0.06, ** *p* < 0.01, *** *p* < 0.001. None of the correlations passed requirements of Bonferroni correction. In IIEF eight subjects submitted, that they were not sexually active in the past 4 weeks, so only SD was measured for them

IIEF abbreviations: *EF* erectile function, *OF* orgasmic function, *SD* sexual desire, *IS* intercourse satisfaction, *OS* overall satisfaction

The particular gender role types (stereotypical masculine, feminine, androgynous, undifferentiated) did not differentiate our sample in any of the measured variables in the Kruskal–Wallis test (all *p* values >0.05), except for extraversion level (*H* = 10.16; *p* < 0.05), which was the highest for the androgynous and lowest for undifferentiated type.

There was no significant difference between pornography users (79 % of the whole group) and non-users (22 %) in the Mann–Whitney test (ps > 0.05) regarding the Big Five trait levels, masculinity, femininity and erectile functions. Differences between these groups were observed for religiosity level, sexual initiation age, number of previous sexual partners and frequency of masturbation. The statistically relevant findings are shown in Table 10.

**Table 10:** The Mann–Whitney test for pornography users and non-users

	Z	*p* value	
Religiosity level	−2.64	<0.01	Non-users < users
Sexual initiation age	−2.18	<0.05	Non-users > users
Number of previous sexual partners	−2.02	<0.05	Non-users < users
Frequency of masturbation	−2.36	<0.05	Non-users < users

All the variables measured in the study were tested. Only significant group differences for *p* < 0.05 were listed. The last column shows relationships of the variable levels between groups. None of the findings passed requirements of the Bonferroni correction

Those men who reported being in a romantic relationship did not differ from the singles in the Big Five features in Mann–Whitney test, or the levels of stereotypical femininity and masculinity (all ps > 0.05). The significant differences were observed for age, EF, IS, OS, relationship quality, condom usage frequency, one’s and partner’s attitudes toward sexuality, satisfaction with sexual life, self-acceptance, sexual encounters, any sexual activity, orgasms and satisfying sexual contacts per month. The findings are shown in Table 11.

**Table 11:** The Mann–Whitney test comparing participants who reported being single or in a relationship

	Z	*p* value	
Age	−2.39	<0.05	In relationship > single
EF	−1.98	<0.05	In relationship > single
IS	−2.83	<0.05	In relationship > single
OS	−2.76	<0.05	In relationship > single
Relationship quality	−3.81	<0.001^a^	In relationship > single
Condom usage frequency	−2.00	<0.05	In relationship < single
Attitude toward sexuality	−2.88	<0.01	In relationship > single
Partners attitude toward sexuality	−2.78	<0.01	In relationship > single
Satisfaction from sexual life	−3.58	<0.001^a^	In relationship > single
Self-acceptance	−2.72	<0.01	In relationship > single
Sexual encounters per month	−3.74	<0.001^a^	In relationship > single
Any sexual activity per month	−3.22	<0.001^a^	In relationship > single
Orgasms per month	−2.45	<0.05	In relationship > single
Satisfying sexual contacts per month	−3.95	<0.001^a^	In relationship > single

All the variables measured in the study were tested. Only significant group differences for *p* < 0.05 were listed. The last column shows relations of the variables levels between groups

^a^Means passing requirements of the Bonferroni correction (*p* < 0.0012500)

The religiosity level did not correlate with any of the investigated variables (measured with Spearman’s rho, all ps > 0.05). The comparison of groups consisted of catholics and non-believers (‘other’ type group was omitted because of low prevalence) has not revealed any significant differences in any of the measured variables (ps > 0.05), except for religiosity level (*Z* = −7.34, *p* < 0.05).

Education level correlated positively with age (*r*_s_ = 0.35; *p* < 0.001), EF (*r*_s_ = 0.24; *p* < 0.05), OF (*r*_s_ = 0.29; *p* < 0.01), IS (*r*_s_ = 0.32; *p* < 0.01), delayed ejaculation (*r*_s_ = 0.22; *p* < 0.05), while negatively with frequency of sexual contacts without penetration (*r*_s_ = −0.21; *p* < 0.05),

Concerning condom usage frequency, besides the observed relationship with Neuroticism, it also negatively correlated with age (*r*_s_ = −0.25; *p* < 0.05) and relationship quality (*r*_s_ = −0.30; *p* < 0.01). Coitus interruptus frequency was also negatively related to frequency of anal sexual contacts (*r*_s_ = −0.22; *p* < 0.05), as well as one’s (*r*_s_ = −0.30; *p* < 0.01) and partner’s (*r*_s_ = 0.29; *p* < 0.01) more negative overall attitude toward sexual activity.

Among the additional findings, it is noteworthy that there seems to be a high compatibility between the participants’ and their partners’ attitude toward sexual activity (*r*_s_ = 0.79; *p* < 0.001), partners’ attitude toward sexual activity and investigated men’s satisfaction with sex life (*r*_s_ = 0.76; *p* < 0.001), investigated men’s attitude toward sexual activity and the perceived quality of relationship with a partner (*r*_s_ = 0.51; *p* < 0.001), partners’ attitude toward sexual activity and relationship with a partner (*r*_s_ = 0.55; *p* < 0.001), relationship with a partner and investigated men’s satisfaction with sex life (*r*_s_ = 0.69; *p* < 0.001).

## Discussion

Before the discussion of our findings relating the Big Five and gender roles we briefly present conclusions from earlier studies. All the data cited from these studies were gathered from age groups comparable to our sample.

Hitherto findings revealed that Neuroticism was associated with higher sexual excitement, sexual curiosity, sexual guilt [3], higher sexual anxiety, sexual depression, sexual self-monitoring (a tendency to be aware of the public impression of one’s sexuality), lower sexual esteem and sexual assertiveness [4], lower sexual satisfaction [7], higher chance of infidelity in relationship [20], marital dissolution and dissatisfaction [21]. Eysenck [3] demonstrated that higher scores on this trait are associated with being more nervous about sexual performance, reporting lower levels of sexual satisfaction, becoming sexually excited quicker but also reacting more intensely with sexual inhibition.

The results of our study showed that Neuroticism was slightly negatively correlated with overall sexual satisfaction (OS) but not related to other erectile functions, or sexual disorder report. The first finding was expected and it remains in accordance with the previous studies [3, 7]. Lack of association with other erectile functions was a bit surprising. On the one hand, generally higher level of emotional instability, anxiety and tendency to experience negative emotions seems to be disruptive to sexual response. This concerns stress reaction, which has an inhibiting impact on sexual expression in various psychological and biological aspects [1]. On the other hand, more neurotic males are generally more emotionally aroused. Thus, some of them may cognitively interpret such arousal in terms of sexual desire, as described in the excitation-transfer theory [22]. Such tendency was presented in previous studies [1] and was found to be stronger amongst younger men. Our findings do not support the notion concerning negative impact of Neuroticism on sexual functioning in young adult males, nor do they directly allow to draw any conclusions of Excitation-Transfer influence on sexual expression. We are more willing to admit that neuroticism has a very individual impact on sexuality of young males. A careful case study analysis could be a useful method to have a closer look on that problem.

Nevertheless, men scoring higher on neuroticism scale in our study were moderately less satisfied with their bodies and slightly less satisfied with themselves as men. These findings show that to some extent neuroticism is linked to the problems with broad aspects of self-acceptance. These aspects are also strictly associated with self-esteem and feeling of being sexually attractive to others. Negative emotionality also affects sex life in a partnership. More neurotic men were slightly less satisfied with the quality of their intimate relationships, they expressed a bit more negative attitude toward sexual activity, and so did their partners. Men scoring higher on neuroticism may have greater difficulties with emotional openness, which is crucial in an erotic situation. This may also affect sexual communication abilities. Problems arising in a relationship may also manifest themselves as sexual problems. However, such concerns are weakly proven by the gathered data. It can be concluded that a higher score on Neuroticism is a risk factor for sexual dissatisfaction but it cannot be construed as definitely destructive per se.

Neuroticism did not correlate with any of the investigated sexual problems. It seems that despite a less positive attitude toward sexual activity, the capability for physiological response like erection and ejaculation remains intact in men, regardless their level of neuroticism [23]. Because we examined a group of 19–39-year-old men, their age may be an important biological factor protecting them from such problems.

While analysing frequency of engagement in particular sexual behaviours, we found that higher scores on neuroticism were related to moderately reduced chance of having anal sex. Apart from consciousness, the impact of which on anal sex was slightly positive, neuroticism was the only Big Five trait that had a decreasing impact on the tendency to get involved in this particular behaviour. We suppose that males scoring higher on Neuroticism scale may interpret the possibility of engagement in anal intercourse as ‘not masculine’ or even ‘homosexual’ behaviour, which may be threatening to their own personal concept of masculinity. As rooted in anxiety, the idea of crossing gender-related borders for accepted, ‘proper’ sexual scripts may result in avoidance of anal sex. Noteworthy is the fact that stereotypical femininity level positively correlated with engaging in anal sex.

It is interesting that we did not observe any association between Neuroticism and stereotypical masculinity and femininity dimensions. We expected a positive correlation with femininity and at least slightly negative one with masculinity. Probably among Polish young adult men a tendency to experience and express negative emotions and emotional instability are perceived as not much related to any gender.

Neuroticism found its reflection in a somewhat increased condom usage but also, comparably, in higher frequency of coitus interruptus. Thus, males with greater tendency to experience anxiety are also more afraid of unwanted pregnancy and, probably, sexually transmitted infections. As the tendency to use condoms should be judged as a positive factor for sexual health, the preference of coitus interruptus is worrisome. This brings a conclusion that more neurotic young men should be given better education about efficient contraception methods.

Generally, the relationships between sexual response, satisfaction and negative (but non-clinical) emotionality (especially anxiety) are complex and ambiguous [1]. In future studies they should be considered in a much broader context. Nevertheless, our findings partially support Eysenck’s hypothesis concerning the role of neuroticism in sexual response [3].

Concerning extraversion, the analysis of previous studies revealed relationships with higher levels of sexual satisfaction [7, 8], sexual permissiveness [24], sexual motivation, but also sexual preoccupation [7], number of sexual partners by age 20, psychoactive substance abuse before or during sexual encounter and an earlier sexual initiation age [25]. One study pointed out lower level of sexual preoccupation and sexual consciousness in more extrovert men [4]. According to Eysenck [3], extroverts, because of their hunger for stimuli, are more likely to experiment with sexual activities, have more sexual partners, or engage in sex more often.

In our study, extraversion was found to be associated with multiple positive aspects of sexual expression. First, it was slightly related to a higher sexual desire and lower rate of erectile dysfunction. This supports findings from previous studies [7, 8]. Extraversion was not associated with any other sexual problems. A modest association with experiencing satisfying intercourses was observed. Mild positive correlations were found with engagement in sexual intercourse, oral sex, orgasm frequency and general frequency of different types of sexual activity per month. These results can be explained similar to the ones from the past studies. Persons scoring higher on extraversion scale are more prone to sensation-seeking activities [3]. Sexual activity of various types gives a possibility to enhance stimulation level by increasing desire, sexual thoughts and behaviours. More extrovert individuals are also more sociable. This is a very helpful characteristic in finding a potential sexual partner. Nevertheless, we did not find a significant relationship with a number of previous sexual partners, as Eysenck suspected [3]. Probably the association of higher extraversion level with a greater number of sexual partners occurs in earlier developmental stage of adolescence [25]. Quite surprising was a lack of any relationship with age of sexual initiation.

More extrovert men seem to be more open to a variety of sexual behaviours, except anal sex. Higher extraversion was found to be moderately correlated with the number of satisfying intercourses and slightly with satisfaction with sex life. Such tendency was also described in previous studies [7, 8]. Although individuals scoring higher on extraversion scale have an increased need for stimulation, there was no association with masturbation rate.

As expected, extraversion correlated with a stereotypical masculinity dimension at a moderate level. Hence, higher extraversion was proven to be an element of masculine gender stereotype.

The subsequent trait was openness to experience. The analysis of the previous findings revealed that its higher levels were correlated with lower scores on sexual nervousness [3], lower sexual anxiety and fear of sex [26], but also greater sexual motivation, sexual monitoring, sexual preoccupation [7], permissiveness [24], higher satisfaction and commitment to intimate relationships [27]. Botwin et al. [28] pointed out that intellectual conversation in a couple seems to be linked to greater love and affection. Being traditional and more conservative (low Openness) was also related to having unprotected sex, having sex and pregnancy (for women) at an early age [25].

In relation to our findings, we observed a positive but moderately low correlation between Openness to experience and the quality of relationship with a sexual partner, partner’s positive attitude toward sexual activity and general satisfaction with sexual life. This result can be accounted by the fact that Openness to experience understood as a preference of novel stimuli is also a tendency to entertain such ‘novel ideas’ [29].

In relation to some usefulness of higher levels of Openness to experience for satisfaction and commitment to intimate relationships [27, 28], this trait may support development of adequate communication skills, which results in keeping accurate level of mutual desire between partners, improves partner’s attitude to sex and the overall quality of the relationship. It may also be linked with lower sexual anxiety and fear of sex [26]. As we found in our study, quality of the relationship with a sexual partner was an important factor of sexual satisfaction (*r*_s_ = 0.69; *p* < 0.001) and higher scores on openness to experience seem to be slightly helpful in gaining higher scores on both of those scales.

It is interesting that none of the sexual behaviours frequency listed in our study correlated with this trait. Engagement in these behaviours and curiosity to experience ‘non-classical’ sexual activity was found to be more closely linked to extraversion (and openness to experience was mildly positively connected with this trait). Likewise, we did not observe any association with erectile functions and sexual problems report.

Earlier studies reveal that low Openness to experience is associated with having unprotected sex [25]. We did not find any relation of this personality trait with condom usage or frequency of coitus interruptus. Neither do our findings support the opinion of higher sexual permissiveness in men who are more open to experience, as it was stated elsewhere [24]. It was not linked to earlier sexual initiation age either.

As expected, openness to experience was mildly associated with stereotypical masculinity, so to some limited extent higher levels of this trait seem to be a part of that gender stereotype.

In other studies, another ‘Big Five’ trait, conscientiousness, was found to be negatively correlated with sexual preoccupation [7, 26], and positively with marital and dating satisfaction [30]. Individuals who scored low on conscientiousness were more likely to have sexual affairs [31], while more conscientious men were found to be more disciplined in delaying potential sexual gratification and avoiding risky sexual behaviours [25].

In our study, conscientiousness was found to be slightly associated with later sexual initiation age. Persons with high levels on this trait describe themselves as well-organised and responsible. Probably, they also postpone moment of starting sexual life with a partner and tend to wait for more ‘proper circumstances’. However, according to our findings, being more conscientious affected having a bit more frequent sexual intercourses, even anal sex and ‘any sexual activity’ with their partner. It is quite interesting that more conscientious males, who were found to be more able to delay potential sexual gratification [25], generally have more sexual intercourses than those with lower levels of consciousness. It is noteworthy that males scoring higher on consciousness scale were mildly less willing to engage in sexual activities without penetration.

More conscious males also declared more satisfying sexual activities per month and better overall sexual satisfaction. This finding remains in accordance with previous reports [30]. In our sample conscientiousness did not affect any of the direct aspects of a relationship with partner.

A moderate association of conscientiousness with satisfaction with one’s body was also indicated. This tendency seems to have a good influence on general sexual expression.

In our study higher levels of conscientiousness moderately decreased the number of masturbation acts. People with this characteristic may hypothetically perceive masturbation as inappropriate, especially if they are concurrently involved in a relationship with a sexual partner. For more conscientious men, following norms is crucial, while for many Polish men masturbation is perceived as morally inappropriate [14]. Although there was no correlation between conscientiousness and condom usage frequency, there is a noticeably decreased tendency to engage in coitus interruptus. This may partly confirm previous findings [25] that men scoring higher on conscientiousness are also more responsible in sexual activities they take part in.

In our sample, higher conscientiousness was surprisingly a slightly protective factor in case of premature ejaculation problems. We expected that an increased tendency to control one’s behaviour causes the opposite effects.

Conscientiousness was found to be moderately correlated with the stereotypical femininity. This can be explained as a culture-induced expectation for women to be more self-controlling and restrained in sexual activity [13]. What is surprising, in our sample of young adult males such ‘femininity’ was slightly associated with better erectile function and orgasm.

The last of the Big Five traits, agreeableness, in previous studies was found to be negatively correlated with sexual motivation and sexual preoccupation [7, 26], positively with marital and dating satisfaction [28, 30]. Partners who scored lower on agreeableness were more likely to have sexual affairs [31]. Low agreeableness was also related to greater number of sexual partners, engaging in risky sexual behaviour and earlier sexual initiation age [25].

We have observed that agreeableness also correlated with various sexual behaviours. There was a weak, negative association with the number of previous sexual partners. Hypothetically, more agreeable men have better partner retention skills, which results in staying in one relationship for a longer time. On the other hand, such men may have a lower interest or lack of skills to engage in short romances or one night stands.

We found that Agreeableness was positively, but mildly associated with having more frequent sexual intercourses and mutual masturbation acts with their partners. Therefore, this personality trait may be helpful in negotiation and gaining partner’s acceptance to initiate sexual activity. Because agreeableness showed a slight negative correlation with frequency of masturbation, it may be a useful trait that helps in reaching a ‘sexual consensus’ with a partner and thus helps to fulfil one’s sexual needs.

It is interesting that we also found a correlation between agreeableness and satisfaction with one’s body. Presumably, men with high levels of agreeableness accept any imperfections of their bodies more easily, or a good relationship with a sexual partner provides a positive feedback about feeling physically and sexually attractive.

Among sexual attitudes, we reported a weak positive association of level of Agreeableness with the quality of relationship with a partner. Such observation is comparable with previous findings [31].

We did not notice any significant association between the level of Agreeableness and erectile functions. In relation to sexual problems, similarly to Conscientiousness, higher scores on Agreeableness were found to be a bit protective against premature ejaculation.

Agreeableness was weakly positively associated with stereotypical femininity and similarly, but negatively with masculinity. The tendency to be cooperative, trustful, moral, altruistic, modest and sympathetic seems to be perceived as more typically displayed by women than men. Such a feminine attitude seems to be useful in dealing with relationship problems and improving a couple’s sex life.

Besides the Big Five traits, we also focused on gender roles’ impact on sexual expression. Some authors [13] state that particular elements of the internalised gender stereotypes should affect sexual expression. As regards the concept of masculinity, it contains a tendency for dominance, aggressiveness, autonomy, self-confidence, staying focused on personal satisfaction or negatively overinterpreting sexual problems. For the femininity stereotype it covers being delicate, sensitive, neat, passive in sex, not very interested in having sexual contacts.

The analysis of data from previous studies has revealed that higher level of masculinity was found to be related to sexual pleasure [32], while other studies suggest that femininity was associated with satisfaction from the current intimate relationship [33] and marriage [34]. Intensity of masculinity was also related to the earlier age of sexual initiation and greater number of previous sexual partners [35]. Among gender role types, androgynous and stereotypically masculine men declared higher levels of sexual satisfaction [32]. Androgyny was also linked to more erotophilic attitude [1, 36].

Extreme levels of internalised masculinity were indicated to be associated with sexual aggression towards women [37]. Nevertheless, in a meta-analysis masculinity measure based on Bem’s gender schema Theory has shown small effect size with sexual aggression. It seems that general gender-role traits do not strongly predict sexual violence [37].

According to our findings, there are mild, positive correlations between higher levels of both masculinity and femininity and aspects of better sexual functioning, but with prevalence of positive masculine impact.

Almost one third of the participants were identified as an androgynous type, which could explain a medium correlation between dimensions of masculinity and femininity.

Masculinity was weakly to modestly associated with a better erectile function and higher sexual desire, while femininity with orgasmic function. According to this data, we could speculate that both, masculine and feminine dimensions, are somewhat necessary and helpful in different aspects of male sexual functioning. Nevertheless, masculinity was associated with greater number of sexually-favourable aspects, such as: satisfying sexual intercourses, orgasms, sexual activities without penetration, any sexual activity per month, one’s and partner’s positive attitude towards sexual activity, satisfaction with sexual life, lower tendency to experience erectile dysfunction. More masculine men also declared a bit higher number of previous sexual partners. All gathered data support previous findings concerning number of partners [35], and sexual pleasure and satisfaction [32]. Unfortunately, analysing our findings, we were not able to say anything about sexual aggressiveness towards women in stereotypically more masculine men. We can only suspect that extremely masculine men [37] build their relationships with partners basing on domination and submission. This problem should be investigated in future studies.

Masculinity was slightly negatively correlated with age. Some interesting findings about personality development, which could be related to the masculinity intensification change, were reported elsewhere [38, 39]. In brief, such change within masculinity-related traits may be the result of social adaptation, becoming emotionally less volatile and more attuned to social demands. Although we studied a group of men in the same developmental stage, we can expect that there is a difference between 19 and 39-year-old men in their psychological profile. Perhaps, for some men getting older also means becoming more balanced in relation to the stereotypical masculine traits. However, for now it is only a hypothesis, which should be verified in further studies.

Higher scores on femininity scale were mildly correlated with erectile and orgasmic functions, longer ejaculation latency and higher frequency of anal sex. We did not find any associations with satisfaction with the intimate relationship, as it was observed in earlier studies [33], nor any other attitude towards sexual expression.

Neither did we observe any relationship between the level of internalised femininity and masculinity gender stereotypes and sexual problems, condom usage or frequency of coitus interruptus.

In the group comparison of gender role types, we did not notice any significant differences in frequency of sexual behaviour, erectile functions or attitudes toward sexuality. Previous findings of higher sexual satisfaction and pleasure [32, 33] were not confirmed in our sample.

The age of participants in our sample was related to a few sexual characteristics. More mature men were slightly less willing to engage in masturbation, sexual contacts without penetration, use of condoms or oral sex. On the other hand, they declared a bit higher number of previous sexual partners, more frequent anal intercourses and, quite surprisingly, were more satisfied with their bodies. Most of these findings could be explained by a greater chance of having a stable intimate relationship for older males in comparison to younger ones. A tendency to prefer anal over oral sexual activity with age is hard to interpret. What is noteworthy is a lower willingness to use condoms by older males and those in an intimate relationship.

As we reported, there was no relationship between religiosity level and any of the measured aspects of male sexual expression. It seems that religiosity had a very limited influence on young males’ forms of sexual expression. It was thus close to the earlier reports [40], according to which religious attitudes and practices do not fully determine sexual behaviour. Such tendency was found to be more characteristic of men, as opposed to women [14]. For males, religiosity was only related to later sexual initiation and less common sexual contacts outside of marriage [41].

Our study is mostly exploratory. However, it gives an interesting overview of the relationship between personality traits, gender stereotypes and various aspects of sexual expression. We suggest further, more detailed verification of the observed tendencies in the future studies on bigger groups.

### Limitations of the study

First, the study sample is homogeneous and relatively too small to draw any definite conclusions. In our sample, we did not report a normal distribution of the Big Five traits (*p* < 0.05 for all traits in the Shapiro–Wilk test). The group was characterized by an increased level of conscientiousness and lower level of neuroticism and openness to experience than expected in the general population.

Some labels were used in the study to assess sexual behaviours may be hard for interpretation. This concerns, i.e. anal sex or mutual masturbation, which could cover a wide range of different behaviours. If we took under consideration methodological assumption of our study, it would be hard to investigate very specific forms of such behaviours. That is why we decided to construct questionnaire items in a more general form.

Another difficulty was to adequately operationalize sexual behaviours and their measures. We are aware that asking about giving ‘mean score’ of engagement in particular sexual behaviour per month or ‘mean time to ejaculate’ is a big, but necessary simplification. These are quite common problems of such studies [1, 14]. Nevertheless, our findings seem to be helpful in revealing the image of sexual expression among Polish young adults.

It is important to highlight the fact that the issue of sexual expression, attitudes and its scientific exploration is still a taboo for a substantial part of Polish population. That explains the low response rate. Nevertheless, it is similar to other studies based on the questionnaire methods. To make our study as representative for the general population of young adult men as possible we reflected the age structure for this group in our sample. Anyhow, we have to take notice that in relation to general population our results are somewhat limited. Future research should make these issues a priority.

Retrospective character of the study may not reflect the actual sexual behaviours in a longer period of time. However, it describes a dynamic construct of sexuality that may change in time. We used only the IIEF for assessing sexual function. Other scales could also be applied. However, the 15-item IIEF meets the standards in satisfying evaluation of sexual function in men.

Last but not least, the present study was exploratory in nature and its basic conclusions need further support from multicenter studies recruiting larger groups of Polish men and women with different socio-demographic characteristics.

## Conclusion

Big Five personality traits have noticeable, but weak to moderate, influence on sexual behaviour in young adult males. Stereotypical femininity and masculinity dimensions have the same effect. Psychological profile is built by multiple personality traits that jointly affect one’s overall functioning. Similarly, sexual expression is also a result of the interrelation of these traits, not only its separated features.

## Abbreviations

### 'Big Five' personality traits

N: neuroticism; E: extraversion; O: openness to experience; C: conscientiousness; A: agreeableness.

### Bem sex role inventory (BSRI)

F: stereotypical femininity; M: stereotypical masculinity.

### Index of erectile function (IIEF)

EF: erectile function; OF: orgasmic function; SD: sexual desire; IS: intercourse satisfaction; OS: overall satisfaction.

## References

[1] Bancroft J (2011). Human sexuality and its problems.

[2] Boyd DR, Bee HL, Johnson PA (2006). Lifespan development.

[3] Heaven PCL, Fitzpatrick J, Craig FL, Kelly P, Sebar G (2000). Five personality factors and sex: preliminary findings. Pers Individ Differ.

[4] Shafer AB (2001). The Big Five and sexuality trait terms as predictors of relationships and sex. J Res Pers.

[5] Fisher W, Byrne D, White L, Kelley K (1988). Erotophobia–erotophilia as a dimension of personality. J Sex Res.

[6] Snyder M, Simpson J, Gangestad S (1986). Personality and sexual relations. J Pers Soc Psychol.

[7] Heaven PCL, Crocker D, Edwards B, Preston N, Ward R, Woodbridge N (2003). Personality and sex. Pers Individ Differ.

[8] Barnes G, Malamuth N, Check J (1984). Personality and sexuality. Pers Individ Differ.

[9] Costa PT, McCrae RR (1992). Revised NEO personality inventory NEO-PI-R and NEO Five-factor inventory (NEO-FFI) professional manual.

[10] Eysenck HJ, Eysenck M (1985). Personality and individual differences: A natural science approach.

[11] Buss DM, Wiggins JS (1996). Social adaptation and five major factors of personality. Theoretical perspectives for the Five Factor Model.

[12] Bem SL (1981). Gender schema theory: a cognitive account of sex-typing. Psychol Rev.

[13] Brannon L (2002). Gender: psychological perspectives.

[14] Izdebski Z (2012). Seksualność Polaków na początku XXI wieku. Studium badawcze.

[15] Central Statistical office (2015). Concise statistical book of Poland.

[16] Zawadzki B (1998). Inwentarz osobowości NEO-FFI Costy i McCrae: adaptacja polska: podręcznik.

[17] Kuczyńska A (1992). Inwentarz do oceny płci psychologicznej: podręcznik.

[18] Rosen RC, Riley A, Wagner G, Osterloh IH, Kirkpatrick J, Mishra A (1997). The international index of erectile function (IIEF) a multidimensional scale for assessment of erectile dysfunction. Urology.

[19] Puchalski B, Szymański FM, Kowalik R, Filipiak KJ (2013). Ocena zachowań seksualnych mężczyzn w ciągu pierwszych 9 miesięcy po zawale serca. Pol Sexol.

[20] Judge TA, Higgins CA, Thoresen CJ, Barrick MR (1999). The Big Five personality traits, general mental ability and career success across the life span. Pers Psychol.

[21] Kurdek L (1993). Predicting marital dissolution: a 5-year prospective longitudinal study of newlywed couples. J Pers Soc Psychol.

[22] Zillman D, Cacioppo JT, Petty RE (1983). Transfer of excitation in emotional behavior. Social Psychophysiology: a sourcebook.

[23] Janssen E (2011). Sexual arousal In men: a review and conceptual analysis. Horm Behav.

[24] Hendrick S, Hendrick C (1987). Multidimensionality of sexual attitudes. J Sex Res.

[25] Miller JD, Lynam D, Zimmerman RS, Logan TK, Leukefeld C, Clayton R (2004). The utility of the Five Factor Model in understanding risky sexual behavior. Pers Individ Differ.

[26] Snell W, Fisher T, Walters A (1993). The multidimensional sexuality questionnaire. An objective self report measure of psychological tendencies associated with human sexuality. Ann Sex Res.

[27] Shaver PR, Brennan KA (1992). Attachment styles and the Big Five personality traits: their connections with each other and with romantic relationship outcomes. Pers Soc Psychol Bull.

[28] Botwin MD, Buss DM, Shackelford TK (1997). Personality and mate preferences: five factors in mate selection and marital satisfaction. J Pers.

[29] Costa PT, McCrae RR (1985). Manual of the NEO personality inventory: form S and FORM R.

[30] Watson D, Hubbard B, Wiese D (2000). General traits of personality and affectivity as predictors of satisfaction in intimate relationships: evidence from self-and partner-ratings. J Pers.

[31] Buss DM, Shackelford TK (1997). Susceptibility to infidelity in the first year of marriage. J Res Pers.

[32] Grabowska M (2011). Seksualność we wczesnej, średniej i późnej dorosłości.

[33] Pei-Hui RA, Ward C (1994). A cross-cultural perspective on models of psychological androgyny. J Soc Psych.

[34] Kuczyńska A (2002). Płeć psychologiczna idealnego i rzeczywistego partnera życiowego oraz jej wpływ na jakość realnie utworzonych związków. Przegląd Psychologiczny.

[35] Fink B, Brewer G, Fehl K, Neave N (2007). Instrumentality and lifetime number of partners. Pers Individ Differ.

[36] Walfish S, Myerson M (1980). Sex role identity and attitudes toward sexuality. Arch Sex Beh.

[37] Murnen SK, Wright C, Kaluzny G (2002). If, “Boys Will Be Boys”, Then Girls Will Be Victims? A meta-analytic review of the research that relates masculine ideology to sexual aggression. Sex Roles.

[38] Staudinger UM, Kunzmann U (2005). Positive adult personality development: adjustment and/or growth?. Eur Psychol.

[39] McCrae RR, Costa PT, Ostendorf F, Angleitner A, Hrebickova M, Avia MD, Sanz J, Sánchez-Bernardos ML, Kusdil ME, Woodfield R (2000). Nature over nurture: temperament, personality, and life span development. J Pers Soc Psychol.

[40] Lefkowitz ES, Gillen MM, Shearer CL (2004). Religiosity, sexual behaviors and sexual attitudes during emerging adulthood. J Sex Res.

[41] Mark KP, Janssen E, Milhausen RR (2011). Infidelity in heterosexual couples: demographic, Interpersonal, and personality-related predictors of extradyadic sex. Arch Sex Behav.

